# RETRACTION: Influences of Evidence‐Based Nursing Intervention on Pressure Ulcers in Intensive Care Units: A Meta‐Analysis

**DOI:** 10.1111/iwj.70162

**Published:** 2024-12-15

## Abstract

**RETRACTION:** XuY.‐B. , ChenZ.‐Q. , SuX.‐H. , and CaoY. , “Influences of Evidence‐Based Nursing Intervention on Pressure Ulcers in Intensive Care Units: A Meta‐Analysis,” International Wound Journal 21, no. 4 (2024): e14834, 10.1111/iwj.14834.38650426 PMC11035972

The above article, published online on 22 April 2024, in Wiley Online Library (http://onlinelibrary.wiley.com/), has been retracted by agreement between the journal Editor in Chief, Professor Keith Harding; and John Wiley & Sons Ltd. Following an investigation by the publisher, all parties have concluded that this article was accepted solely on the basis of a compromised peer review process. The editors have therefore decided to retract the article. The authors disagree with the retraction.

**RETRACTION:** Y.‐B. Xu , Z.‐Q. Chen , X.‐H. Su , and Y. Cao , “Influences of Evidence‐Based Nursing Intervention on Pressure Ulcers in Intensive Care Units: A Meta‐Analysis,” International Wound Journal 21, no. 4 (2024): e14834, 10.1111/iwj.14834.38650426 PMC11035972

The above article, published online on 22 April 2024, in Wiley Online Library (http://onlinelibrary.wiley.com/), has been retracted by agreement between the journal Editor in Chief, Professor Keith Harding; and John Wiley & Sons Ltd. Following an investigation by the publisher, all parties have concluded that this article was accepted solely on the basis of a compromised peer review process. The editors have therefore decided to retract the article. The authors disagree with the retraction.

